# The voice of evidence

**DOI:** 10.7554/eLife.39915

**Published:** 2018-07-11

**Authors:** Eve Marder

**Affiliations:** 1 Volen CenterBrandeis UniversityWalthamUnited States; 2 Biology DepartmentBrandeis UniversityWalthamUnited States

**Keywords:** Living Science, science policy, careers in science, outreach

## Abstract

In an era in which evidence is being disregarded, scientists need to speak up in support of the pursuit for truth.

The first time I remember literally losing my voice I was in Kindergarten at the age of 5. The teacher took attendance by calling our names and we were supposed to respond with "here". I had laryngitis and my "here" was silent. At the end of roll call the teacher announced that I was absent, so I got out of my chair and walked to her desk to tell her I was there. She was annoyed with me as I gesticulated that I had lost my voice, but she did mark me present. The five-year old me experienced very deeply the invisibility that came with losing my voice. To this day, I still get laryngitis and completely lose my voice every so often. When this happens, my husband laughs while pretending to be sympathetic, because he knows that my voice will soon return. Only once have I had to cancel a seminar because I couldn’t croak out a talk.

As a senior woman scientist, I have felt it part of my mission to encourage my more junior colleagues to "take their voices" as scientists, scholars and citizens in the larger scientific community and in the world. But recently, in the Trump and Brexit eras, I feel that I am shouting into the wind of a cacophony of lost souls. How does one effect change when truth has no currency? How do I counsel postdocs that scientific excellence is more important than Journal Impact Factor when so many of my senior colleagues continue to tell them the opposite? How is it possible that, ten years after we came to understand the fallacies of the Impact Factor as a metric to judge individuals, scientists are still making important decisions based on it? Why haven’t we collectively renounced this slavish obedience to perceived excellence rather than real excellence? Why do we still promulgate the belief that where a paper is published matters more than what it says? How can we criticize politicians for not distinguishing between "fake news" and the truth if our community acts in this fashion?

How is it possible that so many of us have been trained to recognize implicit bias and yet nothing changes? Why is it that many hiring committees, organizers of meetings and teams putting together grant consortia end up with groups dominated by white men from a few elite institutions? How is it possible that when we ask for suggestions for new Reviewing Editors at *eLife*, the suggestions most often are for white North American men, and only when we ask people to recommend women or investigators from Europe and Asia, do those names surface (even though they are invariably the same quality as the white American men who were first suggested)?

I have lived through many times of turmoil, both in science and in the world. I remember Watts burning and the riots of 1968. I remember learning that the US government had interned the Japanese-American community in California. I lived through the end of the McCarthy era, and I crossed into East Berlin when the wall was still there. I remember the Cuban missile crisis, and natural disasters and wars all over the world. I have read enough bad political spy novels to believe that governments often lie to their people. I know that serious injustices have been perpetrated all over the world by governments and individuals. But something feels worse today.

**Figure 1:**
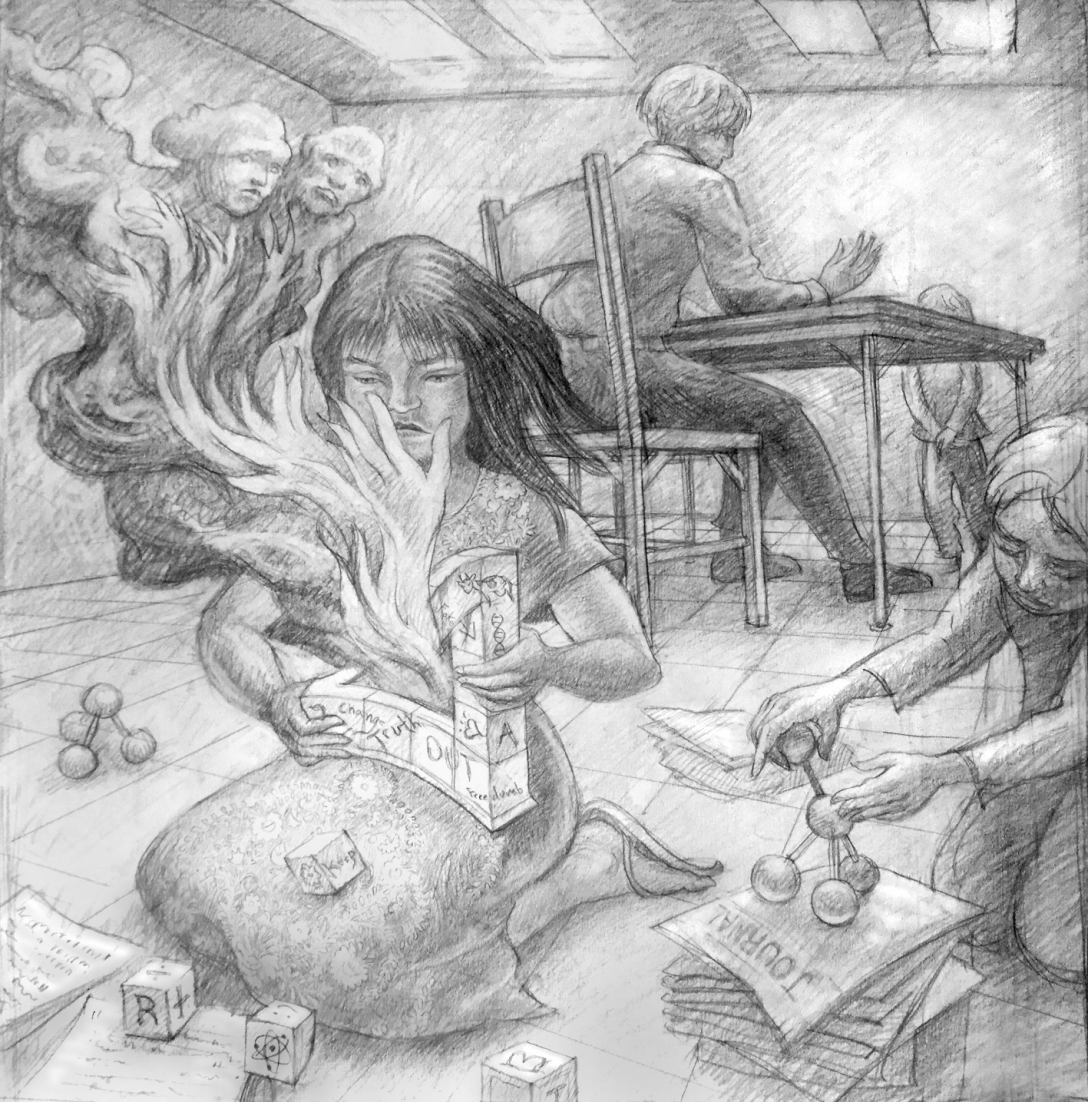
We need many voices, young and old, to remind us that science is about the creation of new knowledge about our universe and its organisms.

Illustration: Ben Marder

In the past, despite everything, I had the faith that the voices raised in the service of "truth" in both politics and science would eventually be heard, and that evidence would be recognized as such. We saw the end of apartheid in South Africa. We saw changes in environmental policy that followed the recommendations of scientists. We saw changes in health care policy informed by science. In that world, our voices could be a reminder that the pursuit of truth in science, in and of itself, was of intrinsic value, and could lead to a better world. The Trump administration’s eagerness to overturn the policies that were made in response to the pursuit of scientific knowledge has shaken me to the core, and I am unconvinced that we will easily recover from the damage done by this president and his disregard for evidence.

The dissemination of new knowledge requires voices willing and able to speak the truth in ways that pierce the clouds of misinformation, misconception, and expediency.

It is still my job to encourage our young to believe in the pursuit of beauty and truth in science. It remains my job to articulate unusual or different scientific perspectives that might change the way we think about some of the fundamental mysteries of the universe. But I increasingly feel that my voice is lost among those treating science as if it were a zero-sum corporate game, even though nothing about the discovery of new knowledge is zero-sum. New knowledge begets the opportunity to beget more new knowledge. The voices describing new knowledge can be drowned by the deafening babbles of our scientific and political worlds. Can we still recognize the voices of those who speak truth? Who, for example, was listening to reports from Puerto Rico about the death toll caused by Hurricane Maria? Where are our senior figures who can speak truth to power and be heard?

Today, more than ever, we need the idealism and drive of our youth. Our young need to sing out with pure voices, raised in the pursuit of truth in science and in life. My voice may be losing its effectiveness, but surely there are others to take my place. But above all, we need many voices, young and old, to remind us that science is about the creation of new knowledge about our universe and its organisms. The dissemination of new knowledge requires voices willing and able to speak the truth in ways that pierce the clouds of misinformation, misconception, and expediency. But, who is listening? Can we successively train our youth to distinguish between truth and falsehood? If not, we are at risk of training a new generation unused to recognizing truth as derived from evidence. How do science and society survive without belief in evidence?

